# Cross-sectoral integration can support reproductive choice, violence reduction and social cohesion: experiences from a climate-stressed district of rural Uganda

**DOI:** 10.3389/frph.2026.1781586

**Published:** 2026-03-25

**Authors:** Susannah H. Mayhew, Alethea Osborne, Dana Kusnir, Gift Namanya, Ruth K. Kisuza, Claire Nimusiima, Richard Muhumuza, Manuela Colombini

**Affiliations:** 1 Department of Global Health and Development, London School of Hygiene and Tropical Medicine, London, United Kingdom; 2 Independent Consultant, London, United Kingdom; 3 Educate! NGO, Kampala, Uganda; 4 Medical Research Council/Uganda Virus Research Institute and London School of Hygiene and Tropical Medicine Uganda Research Unit, Entebbe, Uganda

**Keywords:** climate-stress, cross-sector programmes, qualitative methods, reproductive rights, Uganda, environmental stress, intimate partner violence, integration

## Abstract

**Introduction:**

Climate and environmental stresses have a disproportionate impact on women and girls, acting as a trigger for intimate partner violence (IPV) and compromising access to needed sexual and reproductive health and rights (SRHR) services. Little is known about the impact of cross-sector programmes and services, and programmes responding to these linked crises remain siloed. This paper examines the effect of a novel partnership between environmental protection and livelihoods NGOs and government health services providing integrated programmes in a climate-stressed rural district in Uganda.

**Methods:**

Qualitative research examined whether and how integrated programmes could lead to improvements in IPV, SRHR and wider gender equity in eight villages in Rukiga, Uganda. Forty-four focus group discussions in integrated and non-integrated (“parallel”) sites gathered lived experiences comparing pre- and post-intervention between April 2021–December 2023.

**Results:**

Over time, although participants still described food insecurity, poverty and IPV (prevalent at baseline) many also reported positive shifts in IPV-triggers including gender-dynamics and male attitudes towards family planning (FP). Overall, participants in integrated sites reported increased male acceptance of family planning—reducing a potential IPV trigger - and greater community support for women's economic participation. Integrated delivery that engaged all adults in livelihoods as well as reproductive health activities helped disrupt gendered information (and practice) silos and promote greater reported community cohesion supporting women's legitimate participation in economic activities alongside better male support for women's reproductive rights.

**Discussion:**

Findings suggest that integrated programmes pairing climate-resilient livelihood strategies with rights-based family planning and reproductive health services can enhance reproductive choice for women and help address root-causes of IPV, reproductive coercion and abuse by partners. Protecting women's reproductive rights and wider social wellbeing in the context of severe climate stress means health services and systems must move beyond siloed SRHR programming and service delivery, to embrace partnerships with organisations tackling linked livelihoods and wider wellbeing issues. To be effective, health services and systems must explicitly embed gender-responsive and justice-oriented approaches that ensure the safety of women while addressing persistent inequalities, resource scarcity, and power dynamics.

## Introduction

Climate and environmental stresses have a disproportionate impact on women and girls (1). They exacerbate existing inequities in the distribution of power and resources (including food, water, land, education, healthcare), and in gender inequities, which mean that women and girls are more vulnerable to the ecological and financial disruptions caused by climate variability (2, 3, 52–54). Moreover, poverty, livelihood stresses and food insecurity are known triggers for intimate partner violence (IPV) as well as compromising access to needed sexual and reproductive health and rights (SRHR) services (4–6). Violence against women (VAW), including IPV, is both a cause and consequence of poor health and weakened health systems, particularly in climate-stressed settings where services are already strained (7). Despite these interconnections, few programmes have sought to provide interventions that jointly address climate, environment and livelihoods needs alongside SRHR healthcare, including IPV services.

One partial exception is the population, health and environment (PHE) approach to development programming, defined by USAID as “population, health and environment interventions that are conceptually linked and operationally coordinated at the field level” (8). Based on the premise that rapid population growth, poor health, and environmental degradation are interrelated challenges, PHE programmes seek to provide quality SRHR care alongside livelihoods that are environmentally sustainable and resilient to climate change, often promoting organic farming and agroforestry. The SRHR component, however, did not usually (if ever) include IPV services. Nevertheless, the literature on PHE interventions notes many “value added” benefits of this integrated approach to programming, particularly for programme participants themselves. Benefits included increased community buy-in to SRHR services, improved health outcomes, improved access to and use of contraceptives and increased male involvement in family planning (FP) (9, 10)—all of which may also have a positive effect on reducing IPV. Emerging evidence suggests that integrated PHE and similar multi/cross-sectoral programmes may influence some underlying risk and protective factors for IPV (e.g., by improving women's economic security, decision-making power, and access to SRHR services), yet they have rarely been designed or evaluated with IPV prevention as an explicit primary outcome (11, 12), including protection from reproductive coercion (13).

Actual best practices in cross-sector programme design and delivery remain undefined (8) and have included both coordinated but separate sectoral programming and fully integrated programming offered by multiple cross-sectoral organizations. Further, many reviews have pointed out a dearth of evaluations or studies comparing PHE projects to single sector interventions and have identified the need for additional comparative research (8, 10, 14). Moreover, there is a concern—voiced particularly by some in the reproductive justice movement—that PHE approaches may encourage a population control perspective through their connection of environmental protection with family planning programmes (15). Cross-sector PHE programmes bring into play (and sometimes into tension) three complex elements: reproductive justice (women's right to reproductive choice and autonomy); climate justice (the poorest—particularly women—are disproportionately affected by climate stress yet have done least to cause it); and health systems and service delivery (which must be climate-resilient in order to safeguard SRH healthcare).

Struggles for “reproductive justice” and “climate justice” both have US-based roots and a shared desire to address complex systemic power inequities which intersect with racism, sexism, colonialism, and an uneven distribution of resources (13, 15). Reproductive justice presents a wide understanding of reproductive health and rights, which includes not only the right to have children, but also the right not to have children, and the right to parent in safe and sustainable communities (13). A safe and sustainable community, in this sense, must be free from gender-based violence, including IPV and other forms of violence against women. This includes freedom from reproductive coercion and abuse (the pressure, threats, or manipulation around contraception, pregnancy, and childbearing), which is both a violation of SRHR and a form of VAW (13, 16, 17). Reproductive justice also pushes beyond simply the idea of being “pro-choice” due to an understanding that “choice” is only available to those able to access differing options (55). Climate justice is grounded in the understanding that climate benefits and burdens are unevenly distributed, with those who are least responsible for producing emissions often disproportionately affected by the results of them (15). This has primarily been done at a state or regional level with Climate justice activists fighting for a wider policy framework, including climate financing, which provides appropriate support to those nation states, such as Pacific Island states, disproportionately affected by climate change—support that includes funding for “population” programmes (55). Both movements present important opportunities for framing how to respond to the needs of marginalised individuals, such as rural women, with an intersectional and historically-aware lens. However, while there are shared ideals of justice and equity between the movements, there are also challenges in their integration because of the way reproductive health and environmental resources have been intellectually linked in the past, which at times has led to significant violations of human rights (18, 19).

Climate-stress disrupts health systems functioning and affects quality and coverage of care, particularly reproductive, maternal and child healthcare in low- and middle-income countries (20–23). Interdisciplinary approaches to tackle both immediate stresses and long-term impacts are urgently needed (24, 25), both to build healthcare resilience and to protect and fulfil sexual and reproductive rights (26, 27), including through effective violence against women (VAW) prevention (28). Sexual and reproductive rights are largely absent from literature on health systems responses to climate change challenges; and despite much talk of achieving “equity” and “universal health coverage” through resilient health systems, explicit discourse on justice is also lacking, yet this could bring much-needed urgency to the advancement of women's rights through climate-resilient health systems.

The objective of this study is to consider whether and how integrated programmes could lead to improvements in IPV, SRHR and wider gender equity. To do this, we draw on qualitative insights to examine the contemporary lived realities and experiences of people in climate-stressed Rukiga, Uganda, and the complexities and opportunities provided by this context.

## Methods

### Study setting

“The Healthy Wetlands for the Cranes and People of Rukiga, Uganda” project (henceforth “Healthy Wetlands”) was jointly designed and implemented by the Margaret Pyke Trust (MPT, a UK based NGO), the International Crane Foundation's Uganda branch (ICF), Rugarama Hospital (a local Ugandan hospital) and the London School of Hygiene & Tropical Medicine (29). The project focused on reproductive (and other) healthcare, livelihoods, and environmental conservation. Data collection for formative (baseline) research, process evaluation and endline impact studies took place between June 2021 and December 2023, with the programme ongoing into 2024.

Rural Rukiga encompasses the Rushebeya-Kanyabaha wetland (nesting ground for the endangered Grey Crowned Crane, Uganda's national bird) and extensive farmland. It has seen increased subsistence farming due to population growth and poverty, leading to wetland destruction compounded by environmental changes. Access to family planning and basic health services is poor.

### Study design and programme interventions

A two-armed, quasi-randomized, mixed methods study was undertaken to assess the impact of a cross-sector programme implementing environmental conservation, climate-resilient livelihoods and health (especially reproductive health and family planning) service delivery. Health, livelihood and conservation interventions that were delivered in a fully integrated manner (integrated sites) were compared, over time, to interventions that were delivered separately in a siloed, traditional single-sector approach (parallel sites).

Eight parishes (sites) around the wetland of Rukiga district were assigned to either the “integrated” arm or “parallel” arm. In all sites ICF partnered with community groups to provide a range of livelihoods interventions responding to climate-stresses faced by villagers. These included planting Napier grass and digging water-trenches to reduce soil erosion and building compost heaps to improve soil fertility. Each site also received a range of reproductive and other healthcare services from Rugarama hospital (through outreach services to villages and extra support to static government health facilities), including free family planning services, IPV screening and referral for specialised services for victims/survivors of IPV. Peer educators and Village Health Teams (VHTs) sensitised communities on healthcare issues including reproductive choices (and in integrated sites also on environment and livelihoods issues). The difference between the arms lay in the delivery mechanisms.

In integrated sites, health, conservation, and livelihood messaging and services were delivered together. Thus, health and conservation teams led community events together, with nurses discussing reproductive health and family planning alongside conservationists discussing livelihood and environmental interventions, highlighting the interconnected nature of issues and solutions. In parallel sites, sensitisations and services were provided completely separately by ICF (conservation/livelihoods) and Rugarama hospital and MPT (health), with no cross-referencing of activities or messages and no joint activities or messages.

These interventions were designed to address the challenges identified in the baseline findings [see (29) for detailed description of the interventions and how they were developed]. Baseline data [see (30)] showed that food security was people's top concern, driven by a growing population and limited farmland. IPV emerged as a widespread issue alongside inadequate access to quality family planning services. The research also highlighted complex links between income generation, gender and reproductive health. In response, the project delivered the suite of interventions described above.

### Data collection methods

Across the eight sites, 28 FGDs were conducted at the baseline stage (providing formative inputs to guide intervention design) and 16 at the endline (for impact assessment).

FGDs were conducted by trained researchers who had previous experience in conducting them. Informed consent was secured, and participants were chosen purposively to represent different demographic groups. This included separating men and women in age groups of 18–25 and 26–50, and then a mixed sex group over the age of 50. All the FGDs were conducted in the local Rukiga language, consisted of 6–8 participants and lasted between 1 and 2 h. FGDs were held in settings such as churches or school buildings within the study villages, which were comfortable and safe for both the researcher and participants. Discussions were recorded and subsequently translated into English during the transcription stage. Translated transcriptions were then cross-checked by the other facilitator. Each FGD was designed to capture participants' perceptions, experiences, and challenges within their local communities, and topics ranged from discussions of health services (including FP), environmental degradation, livelihoods, and related social (including IPV) and environmental issues.

### Data analysis methods

Translated and anonymised focus group transcripts were analysed using both NVivo 14 (to organise, code and rapidly extract data) and excel sheets for detailed manual analysis of extracted code data.

The data were analysed using both deductive and inductive approaches through an iterative process undertaken by a multidisciplinary team of qualitative researchers and MSc students. The process evolved over time as findings were discussed and data re-coded and re-searched to gain an in-depth understanding of emerging themes.

The first step was for the research team to familiarise themselves with the data, reading transcripts to gain a broad sense of the discussions, the language used, and any differences in tone, structure, and thematic emphasis across FGDs. This familiarisation was important for grounding subsequent coding decisions in a holistic understanding of the conversations, rather than treating them as fragmented data segments.

An initial set of codes were developed by the research team using four transcripts: one baseline and three endline of which one was from a parallel site and two from integrated sites. Codes focused on community perceptions of integrated programming, its perceived impacts and changes over time, specifically relating to gender roles, IPV, fertility and family decision making and wider women's health and wellbeing issues. These initial broad codes were then applied to the remaining transcripts using Nvivo14. This first round of high-level coding generated two distinct clusters of broad thematic areas: (1) Perceptions of the integrated programme and its effects: Knowledge and perceived benefits of the partner organisations; attitudes towards programme activities; perceptions of change in communities (including health, FP, livelihoods, alcohol use, spousal cooperation, IPV, nutrition); wider and sustained community effects (including perceptions of cohesion, exclusion, sustainability); (2) Perceptions of and attitudes about the issues being addressed by the programme: Connection between health and environment' (with sub-code “connection between family planning and environment”), “gender roles” (with sub codes “access to resources” and “GBV/IPV”), “having many children”, and “women's health services” (with sub-code “family planning”).

Once the initial coding was complete, coded extracts were exported from NVivo into an Excel spreadsheet by theme. In the second phase, (sub)coding was then undertaken manually in Excel, allowing for detailed comparison of coding data between sites and between baseline and endline. Sub-coding focused on identifying patterns, nuances, and contradictions within each broad theme, such as differences in how change was described, variations by participant characteristics, and shifts in language and tone over time. The coding framework was refined iteratively through regular team discussions, where emerging patterns were reviewed and coding decisions adjusted. This process continued until the final thematic structure adequately reflected the range and depth of the data.

### Ethical considerations

This study obtained ethical approvals from The LSHTM Research and Ethics Committee (24031), Makerere University School of Social Sciences Research and Ethics Committee (MAKSS REC 10.20.447/CR) and the Uganda National Council for Science and Technology (HS1137ES) approved this study. We obtained informed written consent from all the participants for those who were literate. For participants who were not, a witness confirmed that they had understood the purpose of the study. The witness was somebody who was chosen and trusted by the participant.

## Results

The views and experiences of participants at the pre-intervention baseline have been reported elsewhere (29–31). In this paper we briefly summarise those baseline findings that are relevant to our impact analysis before presenting the results of the endline impact assessment. Our endline data illustrate how participants' perspectives on and attitudes to environment-health-livelihood linkages, family planning, gender norms and IPV varied between integrated and parallel-delivered sites and how they changed over time.

Participants across the eight sites were broadly homogenous in terms of educational level (ranging from none to tertiary, with most obtaining some primary schooling), ethnicity (mainly Bukiga), religion (Catholic or Protestant) and occupation (many farmers, some small-business people and some teachers).

### Pre-intervention perspectives: interconnected life-challenges; fertility and family planning concerns; ubiquitous IPV

Participants described IPV as widespread and normalised. They described it being driven by poverty, male alcohol use, and shifting gender norms (due to increasing economic independence of women) exacerbated by environmental degradation and climate-related stressors which threatened livelihoods and therefore male breadwinning roles (31).

Further analysis for this paper confirmed the widespread understanding of the interconnections between food security, poverty, large family sizes, lack of access to quality FP services, changing gender roles and IPV.

#### Awareness of environment, food security and human health interconnections

At baseline, both men and women clearly articulated an awareness of the strong relationship between environmental conditions, food security and human health. This understanding was often framed as a mutual dependency, but with only a limited sense of autonomy or control. For example, one woman explained:

“For the relationship between health problems and environmental problems, when you are having peace that can lead to you taking care of your trees and there you are conserving the environment but when you don’t have good health and the health services are not available you will not be able to take care of your trees, you will not look after your children and there is a problem on both the environment and our health.” (female, Integrated, baseline)

This quote frames peace, environmental stability, human health, and support for children as all connected but with little sense of control over any of them. Others described this relationship in even more concrete terms by describing how food insecurity driven by climate crises connects to negative health and wellbeing outcomes:

“They are all connected because the environment and health all move together. For example, when you had planted your crops in a wetland and they are flooded, you lack food and hunger affects one’s health.” (female, Integrated, baseline)

These quotes illustrate an awareness of the negative impact of environmental degradation and reinforce a need for the type of integrated intervention that the programme then offered.

#### Large family sizes regarded as perpetuating poverty and hunger

Participants at baseline discussed the impact of large families primarily through a lens of intense economic challenges. Men highlighted the costs associated with raising children, and the pressure this can place on limited land and resources. Participants consistently described the hardship of feeding, educating, and supporting multiple children while in a position of poverty. As one male participant at baseline explained plainly:

“The population increased and this increased on poverty, you have a big family, but the land is not expanding, this has also increased deforestation. The high population due to high birth rates has so much increased on poverty.” (male, Integrated, baseline)

This insight illustrates an understanding of the pressure that larger families place on limited land resources and the damage this can then cause through processes like deforestation. Another participant connected this failure of the land to support an increasing number of large families with an increased likelihood of community discord and violence:

“When we produce many children when we are not able to get what to give them and where to put them, we don’t have the land to give them because we have many families, this results in killing one another.” (female, Integrated, baseline)

The situation was compounded by a clearly articulated lack of access to quality family planning services, with preferred long-acting methods frequently not available:

“… for the women who want to use family planning, you find that at [the] Health Centre, however, no Nurse can insert an IUD in a woman.” (Male, Integrated, Baseline)

These quotes also suggest a desire for methods which can help mitigate both the challenges faced by larger families and limited land.

#### Changing decision-making power, control of resources and labour contributions

At baseline, there were multiple examples where women reported an increase in their participation in decision-making, particularly in significant economic matters:

“There is a change like in our families the husband would decide to sell whatever he likes and the wife wouldn’t say anything, but because of the rights that were given to us now, he cannot sell anything without the wife being present. if the wife refuses it cannot be sold.” (female, Integrated, baseline)

Similarly, some men at baseline described the importance of consultation and shared decision-making:

“In a family, it’s a man as a head who is supposed to decide together with the woman. A man sits together with the woman and decides on how they want their home to be, then take the idea to the children and the family moves on smoothly.” (male, Integrated, baseline)

These testimonies suggest at least some shared decision-making processes between men and women. However, not all respondents shared this perspective. Some men described strategies to maintain maximum control over resources by deliberately limiting women's access. For instance, one male respondent explained that he shifted to cultivating cash crops specifically so that his wife could not use them for household needs. Elsewhere, women reported men stealing from them, frequently to obtain alcohol:

“Men don’t want to work but they want money for alcohol. So when they fail to get the money to take to the bar, they wait until the woman is out of home and they steal harvested food and take it for sale.” (female, Integrated, baseline)

“…mostly the men drink alcohol and then refuse to dig with the wife. If the man finds some beans in the house, he steals them. I see that men need to change and stop stealing the harvests of women.” (female, Integrated, baseline)

#### Alcohol abuse as a driver of violence

Across all sites at baseline, alcohol use emerges as a major cross-cutting driver of marital discord, poverty, and IPV to an extent that is clearly gendered. Men's excessive drinking of alcohol is linked to a neglect of work, depletion of household resources, and a heightened risk of physical abuse towards women and children. It is often a defining feature of how women describe a man's lack of familial responsibility.

“The money that is intended to support the family is often found to have been spent at the bar, leading to conflicts within the family.” (Male, Integrated, baseline)

“You find that the man has no income. He has resorted to spending all his time at the bar. Now, for me, when I cultivate a given garden, I keep saving some money so that when it accumulates, I can also buy some land for cultivation. When the man discovers that you have bought a piece of land, he can nearly beat you to death. He asks you where you got the money from. Did you adulterate for it?” (female, Integrated, baseline)

Furthermore, respondents spoke about how FP-use can trigger violence, particularly if women resort to secret use, indicating how limited women's decision making is in relation to FP choices:

Most women do not tell their husbands about family planning. They just wake at once and go for family planning without telling their husbands … as the man discovers it, they both resort to quarrels. Now, as their colleagues discover this, they also fear joining family planning (mixed, Integrated, baseline)

### Post-intervention perspectives: shifting norms and motivations on livelihoods, reproductive choice and gender

By post-intervention, although experiences of food insecurity, poverty and IPV remained, many participants described more proactive, mutually reinforcing relationships between environment and health actions, and gave more examples of how these connections enabled reproductive choice. Some attributed these changes to having received integrated services that sought to tackle both access to wanted family planning (as well as other SRHR/health services) and livelihood opportunities that tackle gender equity and address some root causes of IPV. Discussions of large families also evolved from being primarily about economic strain toward deliberate management of family size. Participants in integrated sites reporting wider acceptance and use of FP, especially male acceptance (eliminating another potential trigger for IPV). Integrated delivery that engaged both sexes in livelihoods as well as reproductive health activities helped disrupt gendered information (and practice) silos and promote greater reported community cohesion. These in turn supported women's legitimate participation in economic activities alongside better male support for reproductive rights.

#### Environment-SRHR connections: from awareness to action on reproductive choice

The programme provided interventions across environmental protection, climate-resilient livelihoods, SRHR services and other healthcare. An important difference between integrated and parallel sites was in the experience of cross-sector actions. Many people associated the International Crane Foundation with FP sensitisation (as well as livelihoods and environmental work) and Rugarama hospital as being involved in livelihoods and conservation work. Some people regarded the organisations' concern for both livelihoods and reproductive choice—whether following up on crop yields or ensuring satisfaction with and facilitating method switching for a family planning method—as a sign of respect for people's lives:

“I see that these two projects put much respect in us because like others said, when they give you those seeds they follow them up to see whether the seeds yielded or not, and how much did you get from the harvest. […] Rugarama people also follow up because when you have used a [FP] method and you get issues with it they remove it and advise you on what you can use next.” (mixed, Integrated, endline)

This crossing of sectoral boundaries seems to have contributed to a change in the framing of environment and health connections among participants at integrated sites. While at baseline they already had a good understanding of the interconnections, at endline respondents increasingly discussed proactive actions to protect both health—specifically through reproductive choice—and the environment. Many directly attributed this perspective to lessons learned through the integrated programme. One woman described learning about how the connection between health and the environment was reinforced, and how family planning was positioned at a nexus of it:

“They played a skit on both the Crane’s project and the Rugarama team. And they taught us about family planning. Therefore, we learned that when a person controls his birth, he will be able to have a great environment and good health.” (female, Integrated, endline)

Similarly, two male participants highlighted how programme outreach had integrated environmental and health messages. They explained how recipients of these services now consider outreach efforts (done by vehicle) as a trusted way to receive information and guidance on a range of topics, including health, livelihoods, and development:

“ … most times when we see that vehicle, we know it has come for health issues. But now, it has taught us how we can escape poverty. Also, it teaches us about health issues. So, it has changed something big since they are both sides say on health, the livelihood of people, and development.” (male, Integrated, endline)

“I do thank the Rugarama Hospital because even when they come, they go inspect our plantations. To see whether we planted the Napier grass. Also, they visit homes to see how people conduct themselves. Also, they mobilize people and sensitize them on what they should do.” (Male, Integrated, endline)

These quotes illustrate not only an understanding of the integrated services being offered, but also infer how this integrated approach, which includes information on both health and livelihoods, is more accurately responding to the multi-faceted needs of individuals.

#### Increased access to family planning, improved agency in reproductive health decisions … and ongoing challenges

Access to quality FP services (a major programme focus) was reported (by both programme beneficiaries and non-beneficiaries) to have improved across all sites. Participants were happy with the increased availability of FP services through Rugarama and noted, in stark contrast to baseline, that it was easy to change methods or get long-acting methods removed, if needed.

“Services from Rugarama hospital are good mostly on family planning. Before they came, you would go to the clinic to get an injector plan and they ask for three thousand or five thousand. They inject you minus teaching you or advising you. When the method refuses and you go back, they ask for Twenty thousand shillings and sometimes they fail on removing them. But when Rugarama came, we are very comfortable because you know that any time they are coming and you will remove it if it fails.” (Female, Parallel, endline)

Respondents in integrated sites tended to note wider exposure to FP messages, including men and youth:

“They (International Crane Foundation) taught about 100 women and I saw it was good. They taught them about family planning, making trenches, and growing Napier grass.” (mixed, Integrated, Endline)

“They gave us excellent training to go in our communities to sensitize people about family planning.” (Males, Integrated, Endline)

Greater exposure, through integrated programming, seems to have contributed to an increased willingness to discuss and critique the role of big families amongst both sexes. These discussions naturally included the utilisation or normalisation of family planning methods with participants demonstrating an increased reflection on how family size might be managed more deliberately and the value in doing so. One male participant described how programme engagement had challenged previously held gender-norms, particularly among men who traditionally controlled reproductive decisions in the household:

“It changed my thinking in the way that, some of us men would force our wives to give birth to many children. A woman would like to use family planning and a man refuses her. But now Rugarama has taught that, when you give birth to few children, you can take care of them. Today, when I find Rugarama’s car, I can stop it and [also] ask them about the Napier grass.” (male, Integrated, endline)

The comment illustrates a potential shift in attitude with the financial incentive of fewer children encouraging men to “allow” their wives to use family planning—although whether the choice is truly that woman's is unclear. The comment also indicates a growing comfort among men in seeking information about reproductive health—perhaps because the knowledge of the health providers about livelihoods means men also take their health messages more seriously. The speaker references both family planning and agricultural support and knows he can ask people from Rugarama (the healthcare providers) about climate resilient approaches (such as Napier grass to bind soil and prevent erosion). This reinforces the suggestion that the integration of services has been successful in increasing knowledge and support for FP among men as well as improving access to FP services for women.

Despite some continuing negative male attitudes towards FP, the positive support for FP expressed by men at integrated sites was noticeable, with some noting explicitly that the programme interventions had shifted their perspectives. By contrast, views on FP acceptance were more mixed in parallel communities, with no mention of increased male acceptance or decline in births. Again, a noticeable difference between sites was that in integrated sites FP benefits were reported by non-beneficiaries as well.

Amongst women, the shift in how family size and family planning has been framed accompanies an increased tone of agency around FP use and responsibility. One female participant captured this shift when she said:

“We should give birth to children whom we can take care of.” (female, Integrated, endline)

This perspective suggests large families are not inevitable, and that intentionality in reproductive decision-making is both desirable and increasingly possible. Another participant expressed optimism that such shifts were not only individual, but communal and forward-looking:

“Those who produce many children today, do it just because it's what they want and they know they will manage. I am sure in the coming years, everyone in this community will be able to define on the number of children they want to produce and why.” (female, Integrated, endline)

This quote strongly suggests that the rights-based approach of the programme has been effective in ensuring that the uptake of family planning services feels like an individual and autonomous decision. The quote does not suggest whether the desired number of children will be high or low, but simply that it will be chosen. It is important to note, though, that some participants highlighted the limitations of the positive impact family planning can offer, especially for those who already have many children or face wider economic or marital problems. While family planning services may provide options for the future, they do not address issues faced by families in the present. As one man put it:

“Family planning has been recently introduced. For example, like we who have already given birth to them, what should we do? So, what we want is that you who are in a modern way should find us a way to eliminate poverty.” (male, Integrated, endline)

This quote is significant as it reveals the tension between future-oriented solutions and the ongoing realities for families right now. During FGDs, such challenges were often connected to alcoholism, IPV and limited livelihood opportunities. This complexity was echoed in another reflection from the same FGD, where a speaker challenged the assumption that hardship stems solely from the number of children a family has:

“Regarding giving birth to many children and fewer children. For example, I have 11 biological children plus the one I just adopted making a total of 12 children. Sometimes there is a person you find with 1–2 children yet you find him more burdened. Also, you find another one with no child but still, he cries more. So, should I say it’s also caused by children?” (male, Integrated, endline)

This quote illustrates the far wider elements that may impact an individual's experience of their family size, including access to resources, patterns of consumption, and ultimate desire for a family. For some, many children may be manageable, while for others, even a small family feels overwhelming in the face of environmental degradation, land scarcity, or economic instability.

Participants (particularly women) across both arms asked for more male sensitisation on FP and education of married couples to reduce conflicts, reproductive coercion and IPV:

“I would like them to keep sensitising us, especially men. There are times I go secretly and enrol on family planning because there are times when FP leads to family breakdown. The woman goes for FP without the knowledge of the man, and this creates constant conflicts in the family. So, if the man is also educated and understands the burden the women go through, it would help us in our families.” (female, Integrated, endline)

Specifically, some participants regarded reaching men with education on FP through these cross-sector projects as necessary to reduce IPV risk:

“If these projects put emphasis on teaching men about family planning, it will help us. Because it is one way of reducing domestic violence and misunderstandings in families. Because there are men who want to produce many children yet a woman sees that you will not manage to cater for them. Many women use family planning in secret and the moment the man finds out that you are not on, troubles start from there.” (female, Parallel, endline)

#### Integrated programmes disrupt siloed gender norms and contribute to changing gender roles and power dynamics with wider community effects

All sites reported some wider benefits of programme activities, including knowledge about how to farm in a more climate-resilient way, and greater knowledge of FP methods and services. A key difference between parallel and integrated sites, however, is that respondents in integrated sites mentioned community cooperation and togetherness much more often. Once again, this was reported not just by project beneficiaries by also by people who were not direct beneficiaries, suggesting that the effects are genuine. Specifically, participants noting increased unity as people came together for sensitisations, worked in teams to dig trenches, and beneficiaries shared programme resources with non-beneficiaries:

“I see that it has brought cooperation and togetherness among people, especially in line with community meetings. People participate in meetings and work together as a team. So, there is proper cooperation among people.” (female, Integrated, endline)

Endline data suggests some movement towards greater gender equality in decision-making at integrated sites, through encouraging joint discussion and sensitisation:

“They have promoted equality because they both teach the youth, men and women. They don’t segregate mostly when they are sensitizing about family planning. They also teach it to youths and men. In other words, they aren’t one-sided.” (male, Integrated, endline)

This quote hints at the effect of breaking down more traditionally gendered knowledge areas. Another man described a notable shift in understanding the shared responsibilities of family life due to this non-segregated approach during community engagement:

“These organizations have enlightened people. We used to just give birth but when these projects intervened when a woman is pregnant also a man becomes pregnant with school fees… if a man is understanding, he can acquire family planning by himself… This has taught more people. And people are understanding due to those two organizations.” (male, Integrated, endline)

These quotes illustrate the benefits of ensuring both men and women receive the same interventions. Importantly, this means that not only is the information shared more widely but also that a tone is set in which neither man or woman is seen as the more natural recipient or holder of a certain type of information. By including men in more traditionally female conversations around family planning, and women in the correspondingly traditional male dialogues on climate resilient farming techniques, the changes in typical gender roles (e.g., women being breadwinners, men supporting FP choices) begin to be more accepted. Results suggest that such an approach may help to foster an increased and shared sense of responsibility and equality.

Within the shifting appreciation for the lessons learnt from the integrated programme is an increased recognition of the power that comes from being part of a more formal group. However, this does seem to have been perceived as a primarily empowering opportunity for women. Women's membership in savings and self-help groups is valued at baseline and endline as an effective strategy for women to secure resources independently and protect against men's poor spending habits. At baseline, these groups were often described as informal and formed to circumvent men's control over income. By endline, group support was perceived as more integrated into formal community structures, perhaps due to the integrated programme.

Despite reported positive shifts in women's reproductive and financial autonomy, some men at endline expressed resentment at their own perceived loss of authority. They framed women's increased autonomy, such as their ability to call others for help or support, as a challenge to traditional male power. For example:

“Women have much freedom. And because of these cell phones, when a man starts to talk, they call the chairman and he cools the man down. So we men no longer have the powers in our homes.” (male, Integrated, endline)

This illustrates that while changes and shifts in power may be occurring, they are not universally welcomed and may instead generate new areas of tension. Thus, while the data suggests shifts towards greater shared responsibility and decision-making, particularly in economic and reproductive matters, these gains coexist with persistent structural and behavioural inequalities. Greater women's agency can provoke resistance from men who perceive a loss of authority. Indeed, complex and conflicting power dynamics between men and women are present throughout the data. Discussions at endline, across all sites, continue to consistently frame poverty and alcohol as both a cause and a consequence of “marital discord” and IPV. For example, in the following quote, a man at endline indicates that when a man is drunk his attitudes and trust of family planning may change:

“When I drink alcohol but when my wife doesn’t drink alcohol. So, when she tells me we use family planning, I may not want it. Because such a person [i.e., drunk] doesn’t care. More so, even though the woman uses it without a man knowing. Still, at the end, he gets to know. This, therefore, leads to disagreements in marriage hence family break-ups.’ (male, Integrated, endline)

This quote suggests that preferences or negotiations over family planning become increasingly difficult when alcohol is involved and can then lead to secrecy and distrust within the couple over family planning use. As well as affected autonomy in FP-related decisions, accounts of violence at endline are more explicitly tied to men taking control of women's incomes. In some cases, threats of violence when women refuse to hand over resources were reported: this control over resources was sometimes described in terms that clearly constitute economic abuse, a recognised form of VAW:

“ … at times when he [male partner] gets home, he wants to steal even the little the woman has worked for. When the woman refuses, then violence starts there.” (female, Parallel, endline)

The trajectory is therefore not one of steady improvement in terms of gender equality, but of tensions with change occurring unevenly across households and communities, though with a tendency for more open discussion of the challenges at integrated sites.

## Discussion

For individuals living in Rukiga, concerns over land scarcity, fertility, livelihoods, health, and empowerment were deeply entangled. People clearly and organically situated their reproductive decision-making within broader environmental and livelihood pressures. Between baseline and endline a sense of autonomy over fertility choices increased. A shift in framing was evident amongst some participants away from the economic challenges of having children to a suggestion of the chance to choose when to have them, and to being better equipped to provide for them when doing so. Clear differences between integrated and parallel sites indicate that integrated, cross-sector programming can influence reproductive autonomy, disrupting gender power dynamics and readjusting community relations in a context shaped by traditional male control and IPV.

There was no obvious difference between the sexes nor between sites, in the awareness of linkages between climate resilience and health, although there were subtle but evident differences in tone. Women's accounts leaned towards household and caregiving contexts, such as the ability to look after children, grow food, and manage household resources at times of good health and favourable environmental conditions. Meanwhile, men's accounts more often referenced livelihoods and agricultural productivity. These are tendencies rather than stark divides but mirror some of the clearer differences in framing. Gendered differences were also seen in discussions regarding family size, which had a stronger focus on household and familial concerns for women and a more economic focus for men. These differences in framing provide an insight into gender norms around roles and manifestations of power and, most importantly, how they can be altered by exposures to integrated programmes.

Over time, results showed that there were tangible shifts in attitude and practice among respondents at integrated sites vis-à-vis greater male support for FP. There were also subtle but distinct differences in the openness of discussion about the range of challenges faced, including issues of reproductive choice and autonomy. Collectively these seem to point to a distinctly improved social cohesion at integrated sites at endline.

### Male involvement in FP: opportunities and risks

A key difference was the greater use, and increased male acceptance, of FP in integrated sites. Much literature exists concerning male involvement in FP decision making and its role in women's contraceptive uptake. According to Uganda's most recent Demographic and Health Survey (DHS), 62% of married women who use contraception made the decision to do so jointly with their husbands (32). Various barriers and facilitators to male support for FP have been identified by previous East African studies. Barriers include lack of knowledge of and misconceptions about FP, lack of access to services, socio-cultural stigma, male desire for many children, consideration of FP as a women's issue and fear that FP promotes promiscuity (33, 34). Participants at both baseline and endline in our study spoke of many of these challenges, specifically lack of knowledge about FP, poor access to clinics, misconceptions about FP, fear of side effects and lack of male support. Facilitators to male support include greater wealth and education, positive community attitudes, supportive leaders and an economic need to limit family size (34–37). Of these enablers, positive community attitudes, supportive community leaders and an economic need to limit family size are the most germane to this study's context.

The relevance of FP to promoting economic stability within families has been posited as one of the more successful “hooks” for engaging men in FP, as evidence suggests that men are less likely to participate in dedicated FP programming (38, 39). This was certainly upheld in our paper's findings. In integrated sites, participants were exposed to messaging that framed FP as a tool for maximizing the use of scarce economic and environmental resources by having the desired number of children one could comfortably support. Thus the programme leveraged this “hook”.

Since ICF and Rugarama operated as one organization in integrated communities, sensitisations likely attracted men who were interested in conservation or livelihoods who were then also exposed to FP messaging. This unified approach may have encouraged discussions within couples and the broader community, contributing to greater overall acceptance and use of FP, including male support. In contrast, in parallel sites, men had little incentive to engage with FP sensitisation or services, as these were separate from conservation and livelihoods activities. Women likely attended FP services without their husbands, leaving men unexposed to FP messaging. This segregated approach in parallel sites likely resulted in less discussion within couples and the community, as well as lower male support for FP, due to the absence of the economic “hook” used in integrated programmes. Consequently, community-level acceptance and use of FP were less pronounced in parallel sites.

It is important to note, however, that reproductive decision-making in Rukiga occurs in a context of continuing unequal power relations. Our data show that integrated programmes helped create mixed-gender spaces where SRHR was discussed jointly with livelihoods, and thereby increased legitimacy for FP use. However, our data also show continued barriers (e.g., alcohol, economic stress) and possible backlash risks. For some women, therefore, FP negotiations can still be unsafe and those women may rely on covert use; integrated approaches can reduce the need for secrecy for some women, but not all. Programmes should therefore include safe, confidential SRHR options and referral pathways for IPV support (as this programme did).

### Social cohesion and trust promote behaviour change

Another difference seen across integrated and parallel sites was the greater community cohesion that seemed to have been created in integrated communities. Integrated respondents spoke explicitly of a sense of togetherness and made mention of teamwork (to dig trenches, for example) more frequently than did parallel respondents. Though there is no single definition of social cohesion, it can be characterised by “a set of attitudes and behavioural manifestations that includes trust, an inclusive identity and cooperation for the common good.” (40, 41). Schiefer and van der Noll identify six dimensions of social cohesion (42), two of which are useful in understanding the greater social cohesion evident in integrated sites. One is the “social relations” dimension, encompassing social networks and trust. The quantity and quality of social relations and networks are crucial to developing cohesion. It could be that because of the frequent, integrated nature of the sensitisations ICF and Rugarama provided jointly in the community, these were strengthened. The sensitisations likely created critical spaces for engagement where people could come together, discuss ideas and work towards implementing what they had learned, thereby enabling cooperative activities such as trench digging, or discussing FP. Indeed, the greater male involvement in FP noted by integrated site participants could be seen as a manifestation of this strengthened engagement. Parallel site sensitisations, being sector specific, possibly attracted fewer people with less mixing of community members. This meant less strength and diversity in social relations and networks and parallel sites failed to create as much space for community discussion and engagement.

Trust—both in the delivering institutions and among community members—emerged in our data as a key part of social cohesion. Particularly in integrated sites the clear partnership between ICF and Rugarama hospital fostered a greater sense of trust in these organizations as people saw them joining forces to provide more robust efforts to support linked health and livelihoods needs. On an individual level, hearing integrated messaging (at integrated sites) seems to have brought people together around a shared interest in improving wealth and health for their families, despite differences such as status or gender.

Although not prominent in the PHE literature, the idea that social cohesion—including trust—is key to sustainable development initiatives is not new. Literature from across global public health, conservation, economics and sociology reveals a cross-cutting emphasis on social cohesion and its association with positive development outcomes (44–47). Strong social networks facilitate the diffusion of information (44). Indeed, it seems that integrated programme participants showed a higher commitment to promoting family planning and livelihoods actions. This may in turn result in greater long-term adoption of these practices in integrated sites.

### Reproductive choice and autonomy for women

Our findings point to the importance of male involvement and support for FP and wider social cohesion (that provides an enabling environment for male support) in contributing to reproductive choice and autonomy for women. This was the significant achievement of integrated programming. Nevertheless, gains were uneven and could coexist with backlash.

Having access to family planning has often been a critical marker of autonomy, resilience, and power, and the testimonies from Rukiga reinforce this. Reproductive justice is foundationally linked to ideas of choice and autonomy. Historically, such a sense of choice has been primarily limited to white and more wealthy individuals. A wider understanding of the intersecting elements which shape an individual's ability to achieve bodily autonomy is missing from some reproductive health movements (55). As well as ensuring such a crucial intersectional lens is no longer overlooked, it is particularly pertinent that founders of reproductive justice included a focus on the right to not only have a child, if wanted, but to be able to parent it safely and sustainably.

In climate crises, whatever the injustice of their root-causes, it is unsurprising that the desire to control the size of one's family may be particularly heightened. That is not to say that small families will automatically be preferred, but instead that a sense of autonomy and power is wanted. The FGDs from Rukiga reinforce this view. Individuals highlighted that the positive outcome of improved access to family planning is a sense of control and decision making of when, or not, to have children, and the ability to raise them safely. This does not necessarily signal a simplistic cultural change or a rejection of large families. It is also not a total acceptance of family planning use, but rather an emerging space for negotiation and reflection on the pressures affecting individuals. What emerges is a more nuanced framing which recognises the strain that large families can impose, particularly when living in poverty, and an acknowledgement of the agency people seek in shaping their family size. In these findings there is a clear connection to the commitments of reproductive justice to provide contraceptive care which is grounded in the needs of community and justice. Participants in the FGDs acknowledge the impact that increasing populations are having on the environment around them. They suggest that when combined with other services, family planning options can help to increase their chances of providing for the children they want to have. Family planning is not a catch-all solution to people's problems. Without economic opportunity, improved access to land, and broader social support, reproductive decisions are constrained by structural realities. Nevertheless, family planning should be a critical service in all settings.

It is worth noting that the shift in framing of family size amongst the FGDs in Rukiga to something more controllable and potentially positive could simply be the result of the introduction of family planning services during the project. Respondents may also feel obliged to discuss it positively during FGDs. However, the increased number of quotes discussing the ability and right to choose how many children one should have, including among non-beneficiaries, and the lack of quotes which suggest a set prescription, suggests a successful rights-based approach during the intervention. As cited in the results, the clearest expression of this was the woman who said “*I am sure in the coming years, everyone in this community will be able to define on the number of children they want to produce and why”.*

### Implications for reproductive health programming during times of crisis

The complexities and nuances found in the results illustrate that, while much policy and programming continue to treat health (particularly SRHR and IPV) and environment as distinct domains, lived realities reflect an integrated perspective. Siloed and fragmented sectoral interventions are disconnected from peoples lived experiences and therefore fail to address the structural and relational nature of SRHR, IPV and environmental challenges (48). Given this evidence, which is further reinforced by the findings in Rukiga, there is a clear need for an improved understanding of how to integrate services and respond more accurately to the needs of individuals.

Our findings reveal that integrated programmes that pair climate-resilient livelihood strategies with rights-based family planning and reproductive health services can enhance both shared environmental stewardship and reproductive autonomy. While poverty, food insecurity and IPV remained, participants, particularly in integrated sites, described shifts in male engagement with SRHR, and community support. Such shifts may reduce certain triggers for conflict and enable safer navigation of reproductive decisions. However, norm change takes time and not all men may adhere to such sensitisation or behaviour-change. Therefore, it is important to ensure women's safety during programme delivery to better address IPV and reproductive autonomy.

Integrated programming improves overall acceptance and use of family planning, particularly among men. It achieves this through opening up mixed-gender spaces to normalise discussion of reproductive choices and providing livelihood activities together to both men and women. This joint approach fosters a deeper understanding of programme activities within communities, creating wider social cohesion which can lead to better gender equity and realisation of rights in the long-term. In this way, integrated programmes provide an enabling environment for reproductive choice, which is particularly important for women experiencing violence who can be supported to use effective long-acting methods. Specific actions can be taken by practitioners, policy makers and researchers, to better protect women's sexual and reproductive health and rights in the context of climate change. These include:

- Health services and systems should pursue partnership opportunities for cross-sector programming to pair climate-resilient livelihood strategies with rights-based family planning and reproductive health services
- Integrated programs should provide mixed-gender spaces to normalise discussion of reproductive choices and decision-making
- Integrated programs should provide livelihood activities together to both men and women
- Integrated programs must provide safe, confidential, rights-based provision of FP and IPV support and referral as well as attention to economic abuse and male backlash.
- Further research should identify and evaluate successful models for scaling up cross-sector partnerships that embed gender-transformative and justice-oriented approaches
- Donors and policy makers must increase funding and political commitment for cross-sector programming to address interconnected challenges

We acknowledge some limitations to our study. Although we tried to purposively sample FGD participants to ensure a mix of project beneficiaries and non-beneficiaries, some bias may have been present. The level of positive discussion of the programme interventions among our respondents was striking. This may indicate courtesy bias, although the positive views were also expressed by non-beneficiaries (particularly in integrated sites) which does suggest genuine impact. A limitation for the fertility and IPV perspectives is the lack of data on couple decision making, which limits what we can say about family planning decision-making. The programme interventions did not have a specific focus on couples, although some couples were likely to have received messaging together particularly at integrated sites during combined livelihoods/health activities. Couple-interventions may have an impact on relationships quality and eventually on improving communication skills (which are potential IPV reduction mechanisms in other settings: (49, 50), but we do not have data on this.

In conclusion, our study has found that integrated cross-sector programmes can shift conditions for SRHR uptake (through mixed-gender community communication, building trust and social cohesion). Nevertheless, IPV drivers still remain (though reduced at integrated sites) and can persist in times of livelihood-stress. Integrated programmes should therefore be paired with safe, confidential, rights-based provision of FP and IPV support and referral as well as attention to economic abuse and male backlash. Protecting women's reproductive rights and wider social wellbeing in the context of severe climate stress means health services and systems must move beyond siloed SRHR programming and service delivery, to embrace partnerships with organisations tackling linked livelihoods and wider wellbeing issues. To be effective, they must explicitly embed gender-responsive and justice-oriented approaches that ensure the safety of women while addressing persistent inequalities, resource scarcity, and power dynamics.

## Data Availability

The raw data supporting the conclusions of this article will be made available by the authors to genuine researchers, without undue reservation.

## References

[1] Baschieri A Udeh C Yunusa Z Snow R. Women, girls, and the climate crisis: advancing reproductive health and rights and gender equality in climate policies at ICPD+30. Stud Fam Plann. (2025) 56(2):301–16. 10.1111/sifp.70006 40322932 PMC12205725

[2] UN CEDAW. General Recommendation No. 37 on Gender-Related Dimensions of Disaster risk Reduction in the Context of Climate Change, Committee on the Elimination of Discrimination against Women, 7 February 2018: CEDAW/C/GC/37 (2018).

[3] Schulz P Halperin-Kaddari R Rudolf B Freeman MA. The UN Convention on the Elimination of all Forms of Discrimination Against Women and Its Optional Protocol: A Commentary. Oxford: Oxford University Press (2022).

[4] Bradshaw S Fordham M. Double Disaster: Disaster Through a Gender Lens. Hazards, Risks, and Disasters in Society. Amsterdam: Elsevier (2015). p. 233–51.

[5] Desai BH Mandal M. Role of climate change in exacerbating sexual and gender-based violence against women: a new challenge for international law. Environ Policy Law. (2021) 51(3):137–57. 10.3233/EPL-210055

[6] Hatcher AM Page S Aletta van Eck L Pearson I Fielding-Miller R Mazars C Systematic review of food insecurity and violence against women and girls: mixed methods findings from low- and middle-income settings. PLoS Glob Public Health. (2022) 2(9):e0000479. 10.1371/journal.pgph.0000479 36962559 PMC10021293

[7] Stöckl H Sorenson SB. Violence against women as a global public health issue. Ann Rev Public Health. (2024) 45:277–94. 10.1146/annurev-publhealth-060722-025138 38842174

[8] The BALANCED Project. PHE Field Implementation: A Simple PHE Resource Guide/Compendium for Practitioners. Narragansett, RI: Coastal Resources Center (2013). Available online at: https://peopleplanetconnect.org/phe_resource/phe-field-implementation-a-simple-phe-resource-guide-compendium-for-practitioners/ (Accessed February 27, 2026).

[9] Obat E Schaefer K Opiyo M Otieno G Windindi H Omuodo D Identifying client targets for improved mobilization and uptake of integrated family planning and reproductive health in environmental programs in Kenya. Front Glob Womens Health. (2021) 2:559297. 10.3389/fgwh.2021.559297 34816173 PMC8593978

[10] Yavinsky RW Lamere C Patterson KP Bremner J. “The Impact of Population, Health, and Environment Projects: A Synthesis of Evidence.” (2015).

[11] Ellsberg M Arango DJ Morton M Gennari F Kiplesund S Contreras M Prevention of violence against women and girls: what does the evidence say? Lancet. (2015) 385(9977):1555–66. 10.1016/S0140-6736(14)61703-7 25467575

[12] Ullman C Amin A Bourassa A Chandarana S Dutra F Ellsberg M. Interventions to prevent violence against women and girls globally: a global systematic review of reviews to update the RESPECT women framework. BMJ Public Health. (2025) 3(1):e001126. 10.1136/bmjph-2024-001126 40017928 PMC11816861

[13] Weaver EB Gad L Zota AR. Climate change as a threat multiplier to environmental reproductive justice. Semin Perinatol. (2023) 47(8):151843. 10.1016/j.semperi.2023.151843 37839904 PMC10841484

[14] Pielemeier J. Review of Population-health-environment Programs Supported by the Packard Foundation and USAID. Washington, DC: PRB (2005).

[15] Sasser JS. At the intersection of climate justice and reproductive justice. Wiley Interdiscip Rev. (2024) 15(1):e860. 10.1002/wcc.860

[16] Moulton JE Corona MIV Vaughan C Bohren MA. Women’s perceptions and experiences of reproductive coercion and abuse: a qualitative evidence synthesis. PLoS One. (2021) 16(12):e0261551. 10.1371/journal.pone.0261551 34932570 PMC8691598

[17] Thomas HL Bell SO Karp C Omoluabi E Kibira SPS Makumbi F A qualitative exploration of reproductive coercion experiences and perceptions in four geo-culturally diverse sub-Saharan African settings. SSM Qual Res Health. (2024) 5:100383. 10.1016/j.ssmqr.2023.100383 38911288 PMC11190838

[18] Connelly M. Fatal Misconception: The Struggle to Control World Population. Boston: Harvard University Press (2010).

[19] Stephenson J Crane SF Levy C Maslin M. Population, development, and climate change: links and effects on human health. Lancet. (2013) 382(9905):1665–73. 10.1016/S0140-6736(13)61460-9 23849794

[20] Anton B Cuevas S Hanson M Bhutta ZA Langlois EV Iaia DG Opportunities and challenges for financing women’s, children’s and adolescents’ health in the context of climate change. BMJ Glob Health. (2024) 9(4):e014596. 10.1136/bmjgh-2023-014596 38677778 PMC11057322

[21] Ebi KL Luchters S. Invited perspective: most affected by climate change; least studied. Environ Health Perspect. (2021) 129(11):111301. 10.1289/EHP10384 34747631 PMC8575067

[22] Hagen N Khuluza F Heide L. Quality, availability and storage conditions of oxytocin and misoprostol in Malawi. BMC Pregnancy Childbirth. (2020) 20(1):184. 10.1186/s12884-020-2810-9 32223759 PMC7104524

[23] Saulnier DD Hean H Thol D Ir P Hanson C Von Schreeb J Staying afloat: community perspectives on health system resilience in the management of pregnancy and childbirth care during floods in Cambodia. BMJ Glob Health. (2020) 5(4):e002272. 10.1136/bmjgh-2019-002272 32332036 PMC7204936

[24] Dewi SP Kasim R Sutarsa IN Dykgraaf SH. A scoping review of the impact of extreme weather events on health outcomes and healthcare utilization in rural and remote areas. BMC Health Serv Res. (2024) 24(1):1333. 10.1186/s12913-024-11695-5 39487458 PMC11529210

[25] Ridde V Benmarhnia T Bonnet E Bottger C Cloos P Dagenais C Climate change, migration and health systems resilience: need for interdisciplinary research. F1000Res. (2019) 8:22. 10.12688/f1000research.17559.2 32983410 PMC7506192

[26] Ansah EW Amoadu M Obeng P Sarfo JO. Health systems response to climate change adaptation: a scoping review of global evidence. BMC Public Health. (2024) 24(1):2015. 10.1186/s12889-024-19459-w 39075368 PMC11285469

[27] Saulnier DD Blanchet K. Key ideas and future directions for health system resilience. In: Thomas S Fleming P, editors. Handbook of Health System Resilience. Cheltenham: Edward Elgar Publishing (2024). p. 13–28.

[28] Delany A Mathews S Berry L. Preventing Violence Against Women and Violence Against Children: A Toolkit for Practitioners. Cape Town: University of Cape Town (2025). Available online at: Intersections_Toolkit_web_1704.pdf

[29] Beare M Muhumuza R Namanya G Mayhew SH. A process evaluation of a family planning, livelihoods and conservation project in Rukiga, Western Uganda. Health Policy Plan. (2024) 39(Suppl_2):i93–i104. 10.1093/heapol/czae050 39552342 PMC11570833

[30] Muhumuza R Namanya G Orishaba P Uwimbabazi S Mateeka G Aine-Omucunguzi A Connecting environment, health and livelihoods: how community experiences inform integrated programming in Rukiga District, Uganda. BMJ Glob Health. (2025) 8(Suppl 3):e014406. 10.1136/bmjgh-2023-014406 40527530 PMC12183437

[31] Muhumuza R Colombini M Zilstorf P Collee I Namanya G Katongole J Climate change, livelihoods, gender and violence in Rukiga, Uganda: intersections and pathways. medRxiv [Preprint]. (2025). 10.64898/2025.12.01.25338207 PMC13240901 42247448

[32] Uganda Bureau of Statistics. Uganda Demographic and Health Survey. Kampala: Government of Uganda (2022).

[33] Dougherty A Kayongo A Deans S Mundaka J Nassali F Sewanyana J Knowledge and use of family planning among men in rural Uganda. BMC Public Health. (2018) 18(1):1294. 10.1186/s12889-018-6173-3 30477477 PMC6258500

[34] Kabagenyi A Reid A Ntozi J Atuyambe L. Socio-cultural inhibitors to use of modern contraceptive techniques in rural Uganda: a qualitative study. Pan Afr Med J. (2016) 25:78. 10.11604/pamj.2016.25.78.6613 28292041 PMC5324155

[35] Demeke H Legese N Nigussie S. Modern contraceptive utilization and its associated factors in East Africa: findings from multi-country demographic and health surveys. PLoS One. (2024) 19(1):e0297018. 10.1371/journal.pone.0297018 38241359 PMC10798634

[36] Osuafor GN Akokuwebe ME Idemudia ES. Male involvement in family planning decisions in Malawi and Tanzania: what are the determinants? Int J Environ Res Public Health. (2023) 20(6):5053. 10.3390/ijerph20065053 36981959 PMC10048949

[37] Tekakwo A Nabirye RC Nantale R Oguttu F Nambozo B Wani S Enablers and barriers of male involvement in the use of modern family planning methods in Eastern Uganda: a qualitative study. Contracept Reprod Med. (2023) 8(1):49. 10.1186/s40834-023-00251-x 37845730 PMC10577923

[38] Muhumuza C Sileo KM Wanyenze RK Kershaw TS Lule H Sekamatte S Development of a multi-level family planning intervention for couples in rural Uganda: key findings & adaptations made from community engaged research methods. BMC Womens Health. (2023) 23(1):545. 10.1186/s12905-023-02667-8 37865746 PMC10590522

[39] Wambete SN Serwaa D Dzantor EK Baru A Poku-Agyemang E Kukeba MW Determinants for male involvement in family planning and contraception in Nakawa Division, Kampala, Uganda; an urban slum qualitative study. PLoS Glob Public Health. (2024) 4(5):e0003207. 10.1371/journal.pgph.0003207 38820444 PMC11142587

[40] Chan J To H-P Chan E. Reconsidering social cohesion: developing a definition and analytical framework for empirical research. Soc Indic Res. (2006) 75(2):273–302. 10.1007/s11205-005-2118-1

[41] Leininger J Burchi F Fiedler C Mross K Nowack D Von Schiller A Social Cohesion: A New Definition and a Proposal for Its Measurement in Africa, Discussion Paper (2021).

[42] Schiefer D Van der Noll J. The essentials of social cohesion: a literature review. Soc Indic Res. (2017) 132(2):579–603. 10.1007/s11205-016-1314-5

[43] Mehra D Srivastava S Chandra M Srivastava N Laaksonen M Saarinen HE Effect of physical mobility, decision making and economic empowerment on gender-based violence among married youth in India-SAWERA project. BMC Public Health. (2023) 23(1):548. 10.1186/s12889-023-15421-4 36959579 PMC10034242

[44] Eggers Del Campo I Steinert JI. The effect of female economic empowerment interventions on the risk of intimate partner violence: a systematic review and meta-analysis. Trauma Violence Abuse. (2022) 23(3):810–26. 10.1177/1524838020976088 33287669

[45] Garnier J Savić S Cediel N Barato P Boriani E Bagnol B Mainstreaming gender-responsive one health: now is the time. Front Public Health. (2022) 10:845866. 10.3389/fpubh.2022.845866 35903392 PMC9315286

[46] Gupta J Falb KL Lehmann H Kpebo D Xuan Z Hossain M Gender norms and economic empowerment intervention to reduce intimate partner violence against women in rural Côte d'Ivoire: a randomized controlled pilot study. BMC Int Health Hum Rights. (2013) 13:46. 10.1186/1472-698X-13-46 24176132 PMC3816202

[47] Obiagu AN. Do women’s education and economic empowerment reduce gender-based violence in Nigeria? J Int Women’s Stud. (2023) 25(4):12. Available online at: https://vc.bridgew.edu/jiws/vol25/iss4/12

[48] Weiszhar KL Henley P Vezeau N Auerswald H. One health for all: why gender inclusion matters. Lancet. (2025) 406(10502):424–6. 10.1016/S0140-6736(25)01302-9 40683292

[49] Alsina E Browne JL Gielkens D Noorman MAJ de Wit JBF. Interventions to prevent intimate partner violence: a systematic review and meta-analysis. Violence Against Women. (2024) 30(3-4):953–80. 10.1177/10778012231183660 37475456 PMC10845820

[50] Bacchus LJ Colombini M Pearson I Gevers A Stöckl H Guedes AC. Interventions that prevent or respond to intimate partner violence against women and violence against children: a systematic review. Lancet Public Health. (2024) 9(5):e326–38. 10.1016/S2468-2667(24)00048-3 38702097

[51] Rosen JG Mulenga D Phiri L Okpara N Brander C Chelwa N “Burnt by the scorching sun”: climate-induced livelihood transformations, reproductive health, and fertility trajectories in drought-affected communities of Zambia. BMC Public Health. (2021) 21(1):1501. 10.1186/s12889-021-11560-8 34344335 PMC8335992

[52] Thurston AM Stöckl H Ranganathan M. Natural hazards, disasters and violence against women and girls: a global mixed-methods systematic review. BMJ Glob Health. (2021) 6(4):e004377. 10.1136/bmjgh-2020-004377 33958379 PMC8112410

[53] van Daalen KR Kallesøe SS Davey F Dada S Jung L Singh L Extreme events and gender-based violence: a mixed-methods systematic review. Lancet Planet Health. (2022) 6(6):e504–23. 10.1016/S2542-5196(22)00088-2 35709808 PMC10073035

[54] Silliman J Fried MG Ross L Gutiérrez E. Undivided Rights: Women of Color Organizing for Reproductive Justice. Chicago: Haymarket Books (2016).

